# Annotation Error in Public Databases: Misannotation of Molecular Function in Enzyme Superfamilies

**DOI:** 10.1371/journal.pcbi.1000605

**Published:** 2009-12-11

**Authors:** Alexandra M. Schnoes, Shoshana D. Brown, Igor Dodevski, Patricia C. Babbitt

**Affiliations:** 1 Graduate Group in Biophysics, University of California San Francisco, San Francisco, California, United States of America; 2 Department of Bioengineering and Therapeutic Sciences, University of California San Francisco, San Francisco, California, United States of America; 3 Department of Biochemistry, University of Zürich, Zürich, Switzerland; 4 Department of Pharmaceutical Chemistry, University of California San Francisco, San Francisco, California, United States of America; 5 California Institute for Quantitative Biosciences, University of California San Francisco, San Francisco, California, United States of America; Spanish National Cancer Research Centre (CNIO), Spain

## Abstract

Due to the rapid release of new data from genome sequencing projects, the majority of protein sequences in public databases have not been experimentally characterized; rather, sequences are annotated using computational analysis. The level of misannotation and the types of misannotation in large public databases are currently unknown and have not been analyzed in depth. We have investigated the misannotation levels for molecular function in four public protein sequence databases (UniProtKB/Swiss-Prot, GenBank NR, UniProtKB/TrEMBL, and KEGG) for a model set of 37 enzyme families for which extensive experimental information is available. The manually curated database Swiss-Prot shows the lowest annotation error levels (close to 0% for most families); the two other protein sequence databases (GenBank NR and TrEMBL) and the protein sequences in the KEGG pathways database exhibit similar and surprisingly high levels of misannotation that average 5%–63% across the six superfamilies studied. For 10 of the 37 families examined, the level of misannotation in one or more of these databases is >80%. Examination of the NR database over time shows that misannotation has increased from 1993 to 2005. The types of misannotation that were found fall into several categories, most associated with “overprediction” of molecular function. These results suggest that misannotation in enzyme superfamilies containing multiple families that catalyze different reactions is a larger problem than has been recognized. Strategies are suggested for addressing some of the systematic problems contributing to these high levels of misannotation.

## Author Summary

One of the core elements of modern biological scientific investigation is the universal availability of millions of protein sequences from thousands of different organisms, allowing for exciting new investigations into biological questions. These sequences, found in large primary sequence databases such as GenBank NR or UniProt/TrEMBL, in secondary databases such as the valuable pathways database KEGG, or in highly curated databases such as UniProt/Swiss-Prot, are often annotated by computationally predicted protein functions. The scale of the available predicted function information is enormous but the accuracy of these predictions is essentially unknown. We investigate the critical question of the accuracy of functional predictions in these four public databases. We used 37 well-characterized enzyme families as a gold standard for comparing the accuracy of functional annotations in these databases. We find that function prediction error (i.e., misannotation) is a serious problem in all but the manually curated database Swiss-Prot. We discuss several approaches for mitigating the consequences of these high levels of misannotation.

## Introduction

The frequent addition of new genomes into public sequence databases allows for rapid access to sequences from more than a quarter million named species [1], an accumulation of information that is astounding in both its scale and breadth. While these data hold enormous promise for biological and medical discovery, experimental characterization has been performed on only a tiny fraction of the available sequences. Moreover, current technologies, including high-throughput techniques, can be applied to at most a few thousand genes or proteins at a time. As a result, computational methods are required to predict the molecular functions of the millions of protein sequences that have not and cannot be characterized experimentally. For over a decade, the majority of sequences found in public databases have been annotated using computational prediction alone, raising the issue of annotation accuracy and database quality [2],[3].

Two important papers examining genome annotation error in one and three small genomes respectively [4],[5] predicted that at least 8% of molecular function annotations were incorrect. Depending on the definition of function used, Devos and Valencia further suggested that misannotation levels could be as high as 37%. Other large scale [6] and anecdotal studies describe numerous examples of annotation error (see [7]–[11] for some examples). In a recent paper that modeled annotation error in the Gene Ontology database, it was estimated that up to 49% of computationally annotated sequences could be misannotated [12]. Considering the problem from a different perspective, models of error propagation have shown that with sufficient initial error in a database, error propagation can significantly degrade the quality of the annotations it contains [13],[14] and specific examples of error propagation have been noted [15],[16]. Although functional misannotation remains a significant concern [17],[18], an in depth analysis of the prevalence of annotation error in large public databases has yet to be performed.

Concomitant with the growth of sequence data, annotation strategies have become more sophisticated, benefiting especially from the use of multiple orthogonal methods to improve prediction accuracy (see [19] for a recent review). These include taking advantage of co-localization of functionally linked genes [20]–[23], homology-based annotation transfer using phylogenetic and phylogenomic information [24]–[26], and experimental proteomics approaches such as mass spectrometry [27]. Databases of motifs [28]–[30], sequence profiles [31],[32], and ortholog sets [33],[34], are available for use in computational annotation. In addition, multidisciplinary efforts have focused on accurate annotation for the most important model organisms, including *E. coli* [35], yeast [36], mouse [37] and human [38]. With the availability of such resources, we might expect that the problem of misannotation has diminished. However, the most common approach in use today continues to be the assignment of molecular function from the inference of homology followed by annotation transfer [39]–[41]. Thus, a fresh look at the misannotation problem is timely, particularly for primary public databases containing the largest sets of available sequence data.

In this work, we have investigated the prevalence of annotation error in several large public protein databases in common use today. We examined the large archival sequence databases GenBank NR (NR) [1] and UniProtKB/TrEMBL (TrEMBL) [42], which contain sequences primarily annotated using automated methods. Protein sequences associated with the Kyoto Encyclopedia of Genes and Genomes (KEGG) [43], a database of metabolic pathways, were also examined to estimate the degree to which misannotation has been propagated to a secondary database. These results were compared to those for the manually curated protein database UniProtKB/Swiss-Prot (Swiss-Prot) [42], which is often used in computational analyses as a primary standard for annotation information.

Misannotation levels were determined for sequences annotated to the functions of experimentally well-characterized enzyme families and superfamilies used as a “gold standard,” allowing us to identify misannotated sequences with confidence. Except for Swiss-Prot, all of the databases examined exhibited much higher levels of misannotation than have previously been suggested. Examination of the NR database revealed both evidence for error propagation from previously misannotated proteins and that levels of misannotation have increased over time. The major types of misannotations that were found were classified and their prevalence determined, allowing us to propose strategies for addressing some of the problems that contribute to them. This is the first study to use a gold standard set of superfamilies and families to examine misannotation in the archival NR and TrEMBL databases.

## Results

**Figure 1:**
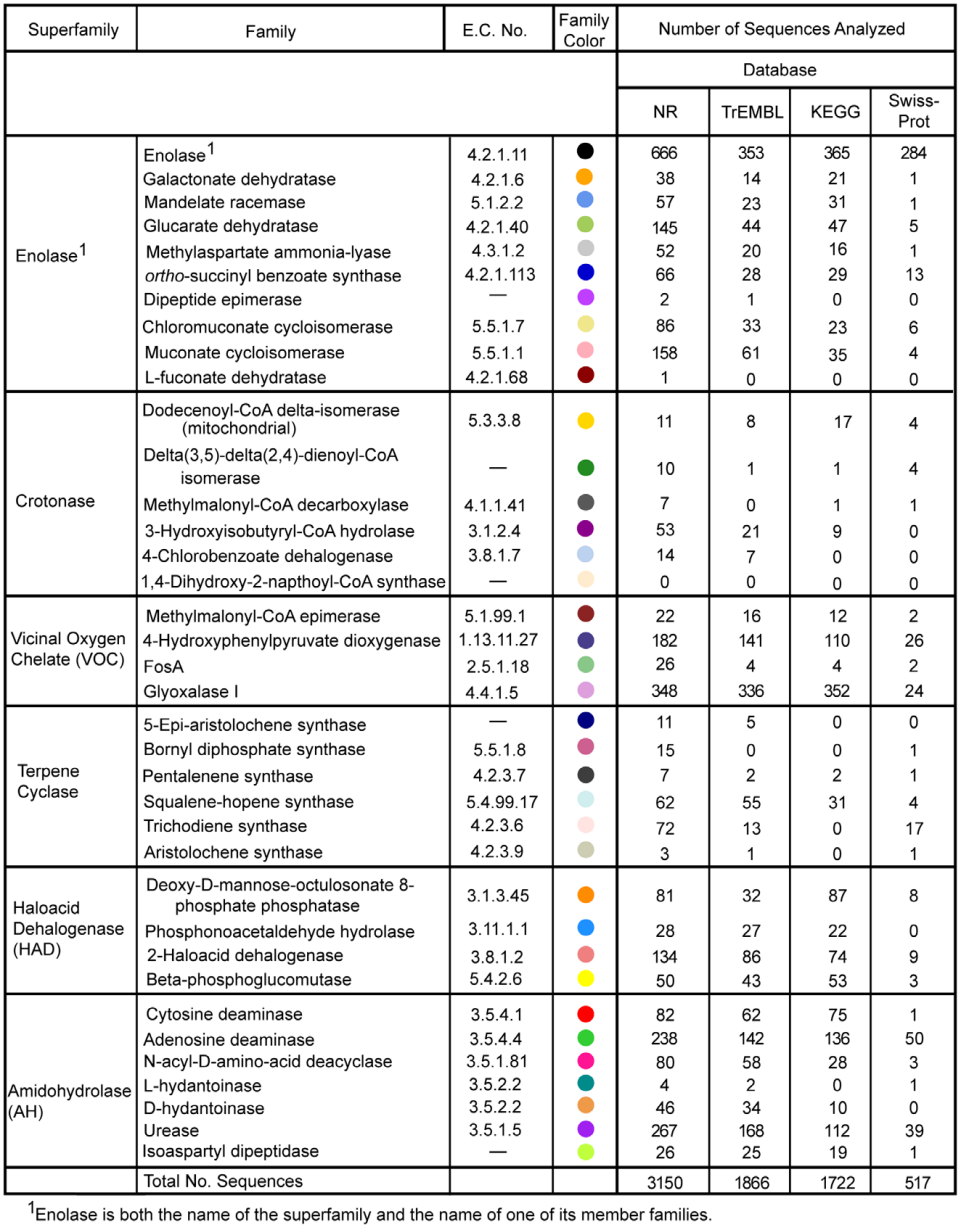
Enzyme superfamilies and their constituent functional families examined in this analysis. Families analyzed in this work are shown organized by the superfamilies to which they belong. Names of superfamilies and families are from the SFLD. E.C. numbers are included where available. Dashes (—) are used for those families for which a full E.C. number has yet to be assigned. Each family is designated by a specific color and these mappings are also used in Figure 3 and Video S1. The number of sequences in each family that were analyzed from each database is listed; the total number of sequences analyzed from each database is also given.

Annotation error in the NR, TrEMBL, KEGG, and Swiss-Prot databases was determined using as a gold standard 37 highly curated and experimentally well-characterized enzyme families from the Structure-Function Linkage Database (SFLD) (http://sfld.rbvi.ucsf.edu/) [44]–[46]. This approach allowed us to achieve an accurate count of misannotated sequences for each family. Enzymes were chosen for analysis because they typically have concrete, precise definitions of molecular function compared to many other classes of proteins. In this work (and as defined in the SFLD), a superfamily is defined as a set of homologous proteins in which conserved sequence or structural characteristics can be associated with conserved functional characteristic(s). A family is defined as a set of homologous proteins within a superfamily that perform an identical function by the same mechanism. These 37 families were chosen because their members have been well characterized by mechanistic analysis and in most cases, x-ray crystallography. They come from six different superfamilies (enolase, haloacid dehalogenase [HAD], vicinal oxygen chelate [VOC], terpene cyclase, amidohydrolase [AH] and crotonase; see the SFLD for references) representing five fold classes and enzymatic functions spanning five major classes of the Enzyme Nomenclature Commission (E.C.) system [47]. (At the start of this analysis the SFLD did not contain any ligases and therefore the sixth major E.C. class, ligases, were not included in this test set.) A total of 7255 sequences annotated to the functions of these 37 gold standard families were evaluated from the four public databases (Figure 1).

**Figure 2:**
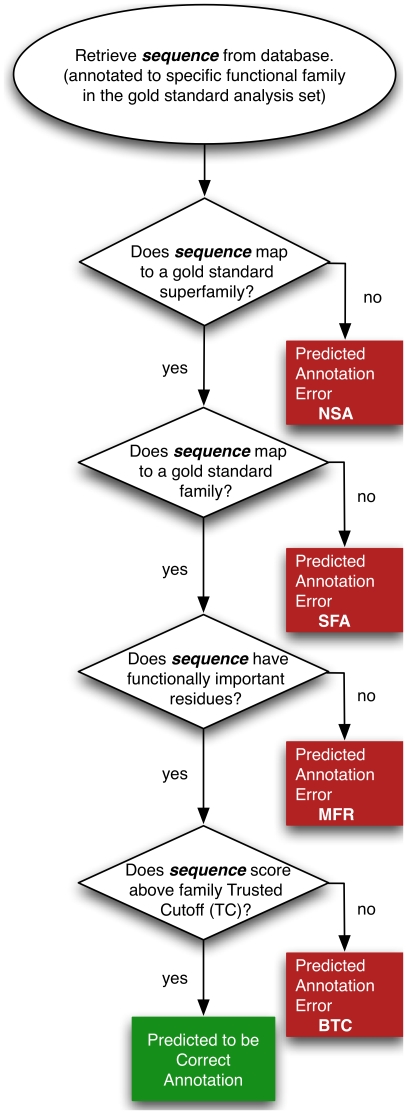
The misannotation analysis protocol. Annotations determined to be incorrect are labelled with the following codes depending on the type of misannotation: ‘No Superfamily Association’ (NSA); ‘Missing Functionally important Residue(s)’ (MFR) ‘Superfamily Association only’ (SFA) ‘Below Trusted Cutoff’ (BTC). See Methods for more detailed discussion of these definitions.

The misannotation analysis presented here examines the question: Given a sequence annotated to a specific enzymatic function, is the annotation correct? Misannotations were identified using sequence, structural and mechanistic information from the SFLD and the literature. Each sequence was analyzed using a four-step protocol (Figure 2) where at each step a sequence could either ‘fail’, be classified as misannotated and labeled with a code defining the type of misannotation, or could ‘pass’ and then be reexamined in the next step. In brief, the analysis steps examined whether the sequence under investigation 1) matched the known sequence patterns of the superfamily to which it was annotated, 2) matched the known sequence patterns of the gold standard family to which it was annotated, 3) contained the residues known to be important for the annotated function, and 4) scored sufficiently well against hand curated hidden Markov models (HMMs) to be considered a member of the annotated family (details in Methods). Family specific cutoffs defined the scores required to confirm membership in each family. Misannotation was defined as the incorrect annotation of a sequence with a specific enzymatic function, determined by its failure to pass any one of these four steps.

**Figure 3:**
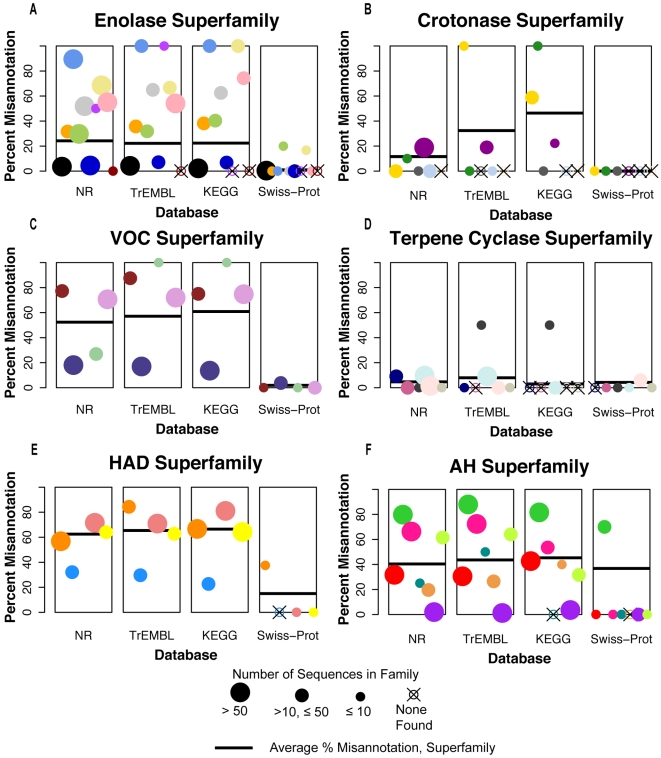
Percent misannotation in the families and superfamilies tested. The results are organized by superfamily: Panel A: enolase, B: crotonase, C: vicinal oxygen chelate, D: terpene cyclase, E: haloacid dehalogenase and F: amidohydrolase. Each panel depicts the percent misannotation for the superfamily in four plots, corresponding to the databases investigated. In each plot, the black bar denotes the average percent misannotation for that superfamily in that database. The percent misannotation for each family within the superfamily is given by a colored circle. The size of the circle provides an estimate of the number of sequences evaluated for that family (scaling in legend). An X through an open circle means that no sequences annotated with that function were retrieved from that database. The order of the families depicted for each superfamily is arbitrary but is consistent through all four plots. The colors of the family circles correspond to those used in Figure 1, which provide a mapping between these family colors and their gold standard functions.

Figure 3 summarizes the results, showing that misannotation was found in all six superfamilies examined (see Table S1 for tabulated values associated with Figure 3). The average levels of misannotation varied greatly between the superfamilies but were remarkably high for four of the six superfamilies (enolase, VOC, HAD, AH) in the three databases NR, TrEMBL and KEGG. The average percent misannotation for these four superfamilies ranged from a little under 25% in the enolase superfamily to over 60% in the HAD superfamily (Figure 3A, C, E, F). In the crotonase superfamily, the average percent misannotation across the superfamily was greater than 20% only in the TrEMBL and KEGG databases (Figure 3B). For five of the six superfamilies, the results for the NR, TrEMBL and KEGG databases were nearly identical (Figure 3A, C–F). For example, in the enolase superfamily (Figure 3A) the average percent misannotations in the NR, TrEMBL and KEGG databases were 24%, 22%, and 22%, respectively. The crotonase superfamily (Figure 3B) was the outlier, showing different levels of misannotation across these databases, (NR [12%], TrEMBL [32%], KEGG [46%]). In contrast, the families in the terpene cyclase superfamily (Figure 3D) were consistently the best annotated with relatively low but still significant levels of misannotation in all four of the databases: NR (5%), TrEMBL (8%), KEGG (3%), and Swiss-Prot (4%).

Across the entire test set, the manually curated Swiss-Prot database was uniformly the best-annotated, showing an average percent misannotation level of 0% (or very nearly 0%) for four of the superfamilies (Figure 3A–D). For all of the superfamilies, misannotation levels were much lower in Swiss-Prot compared to those for the automatically curated databases.

Similar to the results across superfamilies, most of the 37 families investigated displayed consistent levels of misannotation across the NR, TrEMBL and KEGG databases. For instance, the average percent misannotation in the 4-hydroxyphenylpyruvate dioxygenase family (Figure 3C, VOC superfamily, purple-blue dot) was 18%, 17% and 14% in NR, TrEMBL and KEGG, respectively. This result was not surprising given that many sequences within the databases are identical to one another, with identical functional annotations (data not shown). In contrast, large differences in the levels of misannotation were found among the families within a superfamily. For example, in the enolase superfamily (Figure 3A), the family percent misannotation in NR ranged from a minimum of 0% (red dot, fuconate dehydratase family) to a maximum of 90% (light blue dot, mandelate racemase family). As before, variation in the levels of misannotation of families within a superfamily is most pronounced in the databases annotated largely by automated methods (NR, TrEMBL and KEGG). The highly curated Swiss-Prot database showed very low levels of misannotation for the majority of the families investigated; however, even in Swiss-Prot, a few families showed quite high levels of misannotation.

The accuracy of these results was validated using several orthogonal protocols (see Text S1). The literature was searched for experimental results that might contradict our predictions of misannotation. Contradictions were found for only six sequences out of 1155 that had been labeled as misannotated in NR and Swiss-Prot. Another test of our predictions was a blinded analysis of proteins that had been newly experimentally characterized subsequent to our initial analysis. Out of 27 newly characterized sequences, spanning 12 of the 37 families investigated, 26 were found to have been correctly classified by our analysis protocol. We also examined whether annotation corrections had been made for misannotated sequences in the databases since the databases were downloaded for this analysis. Of a random sampling of 111 of the 1112 sequences in the NR database found to be misannotated by our analysis, 96% had unchanged functional annotations. Only for three sequences had the annotations been corrected; one other sequence no longer had a functional annotation.

### Effect of threshold stringency on misannotation levels

The effect on predicted levels of misannotation due to the use of a relatively stringent similarity threshold (Trusted Cutoff (TC)) in the final step of the analysis protocol was evaluated using less stringent thresholds for the NR database. (See Methods for details about how threshold cutoffs were determined.) Two less stringent thresholds for each family were defined, a Noise Cutoff (NC) and Lenient Cutoff (LC), and each sequence was re-analyzed using these thresholds (Figure S1). While the use of these alternative thresholds resulted in somewhat lower levels of misannotation, for most families most sequences identified as misannotated did not change (Figure S2).

### Misannotation over time

**Figure 4:**
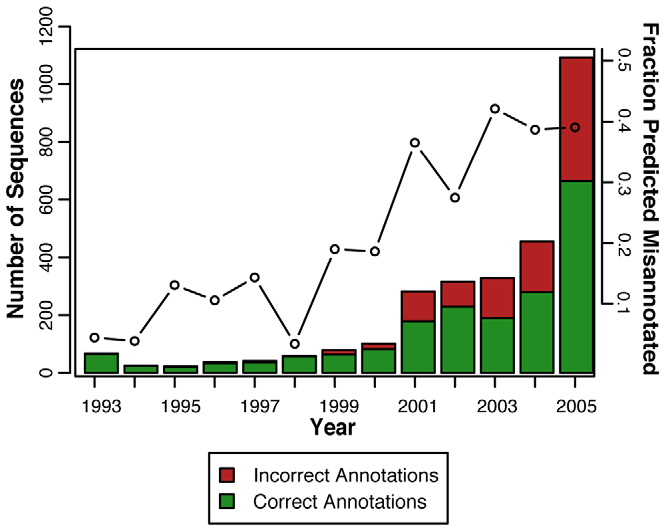
The change in misannotation over time in the NR database for the 37 families investigated. Sequences are plotted by the year when they were originally deposited in the database (x-axis). The number of sequences (left y-axis, bar graph) found to be correctly annotated is shown in green. The number of sequences found to be misannotated is shown in red. The bars for each year represent only the sequences deposited into the database in that year. The fraction (right y-axis, line plot) of sequences deposited each year into the NR database that were misannotated is given by the open nodes, connected by the black line to aid in visualizing the overall trend. This fraction represents the number of sequences in the 37 test families predicted to be misannotated divided by the total number of sequences deposited each year from the test set, i.e. the sum of the sequences depicted in the red and green bars for each year.

Expecting that larger volumes of sequence data and improved methods for annotation would result in higher accuracy annotations over time, we investigated whether the levels of misannotation had changed over the period 1993–2005. Using sequences from the NR database, the original sequence submission dates were retrieved and binned into groups based upon their submission dates and misannotation assignments (“correct” or “incorrect”) according to our protocol. (Because the levels of misannotation for the three automated databases NR, TrEMBL and KEGG were similar, only the NR database was examined in this analysis.) Surprisingly, we found that for the 37 families investigated in this study, misannotation has increased over this twelve-year period: essentially no misannotated sequences were submitted in 1993, while in 2005 approximately 40% of the sequences submitted to NR were misannotated (Figure 4). Not only were more misannotated sequences deposited in the later years, they represented an increasing fraction (black line) of the total depositions annotated to the 37 families. This suggests that the rising level of misannotation is not simply due to the submission of increasingly greater numbers of sequences over this time period, but rather, that the real level of misannotation is indeed increasing.

### Types of misannotation

**Figure 5:**
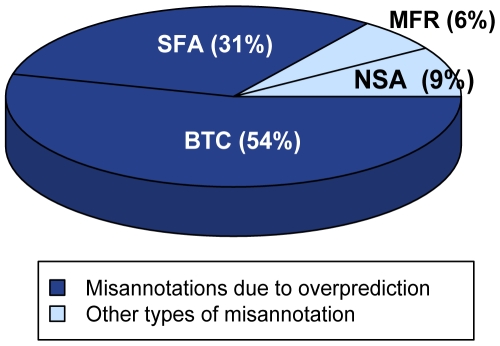
Distribution of major types of misannotation found in the NR database. Classification of misannotated sequences follows the steps of the protocol given in Figure 2: ‘No Superfamily Association’ (NSA); ‘Missing Functionally important Residue(s)’ (MFR) ‘Superfamily Association only’ (SFA) ‘Below Trusted Cutoff’ (BTC), as described in methods. The codes were grouped into two sets that specify whether the misannotation is associated with overprediction or to other types of errors (e.g., missing a required residue).

To better understand the types of misannotation that were found, each misannotated sequence was labeled with an individual, mutually exclusive evidence code describing the type of annotation error it represented. Four primary classes of misannotation emerged from the protocol used in the analysis (Figure 2). Figure 5 shows their distribution for sequences from the NR database. (As with the two previous analyses, only the NR database was examined.) The two misannotation codes ‘Below Trusted Cutoff’ (BTC) and ‘Superfamily Association only’ (SFA) describe cases of overprediction, in which proteins have been annotated to functions that are more specific than the available evidence supports. SFA describes cases in which sequences do not score against the specific family HMM but instead score only against superfamily HMM(s), i.e., HMMs that capture similarities across all families in a superfamily and that therefore do not distinguish families with different reaction specificities. BTC describes cases in which sequences were found to score against both a superfamily and a specific family HMM and contain the necessary functionally important residues, yet failed to score above the TC threshold. Often, this designation refers to a sequence that should have been assigned to a different but similar family, determined by the sequence scoring better against another family HMM. (In these cases, some known functionally important residues may be the same for both families.) The majority of misannotations in the NR database were found to be overpredictions of these two types (85%, SFA + BTC). The remaining 15% of misannotations were associated with the two other misannotation codes, ‘No Superfamily Association’ (NSA) and ‘Missing Functionally important Residue(s)’ (MFR). These codes describe cases in which a sequence could not be associated with the superfamily (NSA), or does not have the necessary functional residues even though it scored against the family HMM (MFR).

Examples of some misannotations from the NR database that were associated with these misannotation codes are provided in Table 1. An example of an NSA misannotation is gi 505585 (GenBank:CAA48717), a sequence from soybean that had been annotated to the glyoxalase I function (VOC superfamily). This sequence did not score against any SFLD HMMs. When searched against the Pfam database [31], the sequence had significant matches only against the glutathione transferase (GST) N- and C-terminal domain models but did not score against glyoxalase I related models. A literature search showed that in this organism, a different gene, gi 4127862 (GenBank:CAA09177), has been characterized as the authentic glyoxalase I enzyme [11]. Further, in the same paper, the gi 505585 sequence had been characterized as a GST, confirming our prediction that gi 505585 had been misannotated. This sequence corresponds to Swiss-Prot sequence P46417 (Swiss-Prot: P46417), also incorrectly annotated as a glyoxalase I.

**Table 1:** Examples of predicted misannotations in the NR database.

Misann. Type	Example	Notes
NSA	**gi**: 48861106 **annotation**: COG1657: Squalene cyclase’ **superfamily**: terpene cyclase	Sequence does not score against any HMMs in the SFLD. In InterPro sequence maps to the carbohydrate binding superfamily and the galactose-binding like superfamily. Sequence does not map to any squalene cyclase motifs [69] or known models.
	**gi**: 505585 **annotation**: ‘lactoylglutathione lyase’^a^ **superfamily**: VOC	Sequence does not score against any HMMs in the SFLD. Sequence matches well against the glutathione transferase N- and C-terminal domain Pfam-A models. Sequence was experimentally characterized and found to be a glutathione transferase rather than a glyoxalase [11].
SFA	**gi**: 17987990 **annotation**: ‘MANDELATE RACEMASE’ **superfamily**: enolase	Sequence does not score against the mandelate racemase family HMM but scores well against the fuconate dehydratase family HMM and contains residues necessary for this function.
	**gi**: 52628216 **annotation**: ‘3-hydroxyisobutyryl Coenyzme A hydrolase’ **superfamily**: crotonase	Sequence does not score against the 3-hydroxyisobutyryl CoA hydrolase HMM but does score against six other family HMMs in the crotonase superfamily.
MFR	**gi**: 17983363 **annotation**: ‘2-HALOALKANOIC ACID DEHALOGENASE I’ **superfamily**: HAD	Asp 180 that is necessary for the hydrolysis of the ester intermediate is substituted with an arginine. Mutational work by Kurihara et al. [70] indicates that this substitution would deactivate the enzyme.
	**gi**: 71915096 **annotation**: ‘n-acylamino acid racemase : O-succinylbenzoate-CoA synthase’ **superfamily**: enolase	Sequence has a histidine at position 166 along with several other substitutions to canonical OSBS/NSAAR motifs.
BTC	**gi**: 16082480 **annotation**: ‘Galactonate dehydratase’ **superfamily**: enolase	Sequence hits the galactonate dehydratase family HMM (bit score: 126.6), but this score is well below the trusted cutoff for the family (TC = 843.6). Sequence does score against the gluconate dehydratase^b^ family HMM with statistical significance.
	**gi**: 56604528 **annotation**: ‘adenosine deaminase’ **superfamily**: AH	Sequence hits the adenosine deaminase family HMM (bit score: 9.7), well below the trusted cutoff for that family (TC = 645.9). Sequence does score significantly against an AH superfamily HMM in the SFLD for sequences of unknown function.

a Synonym for glyoxalase I family function.

b This family was not in the gold standard analysis set because not enough experimental information was available at the time this analysis was performed.

**Figure 6:**
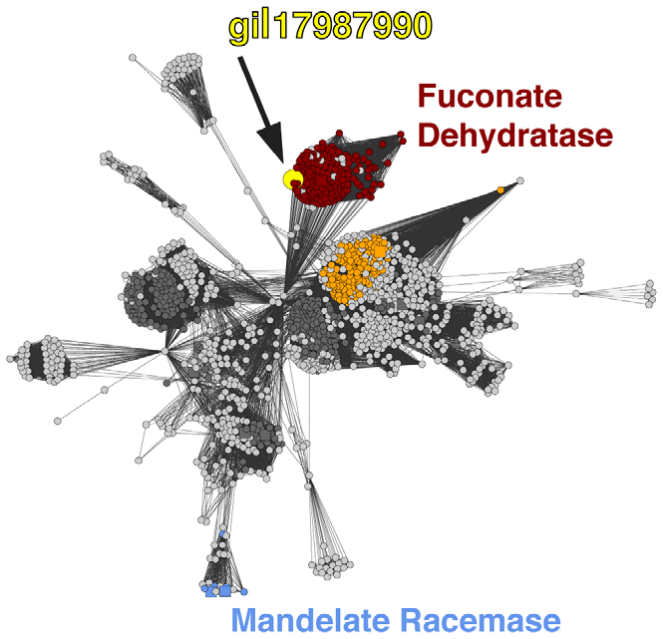
Network view of a misannotated sequence. The protein similarity network shows clustering of sequences from an all-by-all BLAST analysis of a subgroup of the enolase superfamily. Light grey nodes (circles): unknown function; dark grey nodes: sequences annotated in the SFLD but not examined in this analysis; colored nodes: sequences colored by SFLD annotation (as designated in Figure 1, enolase superfamily). Squares represent proteins that have been experimentally characterized and colored circles represent those in which residues known to be important for function and other characteristics for that specific family are conserved. Edges (lines) show BLAST connections between sequences that have an E-value at least as good as 10^−50^. Lengths of edges indicate that sequences in tightly clustered groups are relatively more similar to each other than sequences with few and distant connections. The sequence annotated in GenBank as a mandelate racemase (gi|17987990, yellow dot) clusters with fuconate dehydratases (red cluster) suggesting that it should be annotated as a fuconate dehydratase instead of as a mandelate racemase. The blue cluster containing two characterized mandelate racemases is not close to the fuconate dehydratase cluster, providing further evidence that this sequence is not a mandelate racemase.

An example of an SFA misannotation is gi 17987990 (GenBank:NP_540624), annotated to the mandelate racemase function in the enolase superfamily. This sequence did not score against the mandelate racemase family HMM, but it did score against other enolase superfamily HMMs. In particular, it scored above the TC for the fuconate dehydratase family and contained all the necessary functional residues for that function. As such, we predicted that this sequence is misannotated and that it instead catalyzes the fuconate dehydratase reaction. Using gi 17987990 as a query, 11 other sequences in NR score against this sequence with a BLAST E-value of better than or equal to 1×10^−150^ and are also annotated as ‘mandelate racemase,’ likely indicating a case of error propagation. A protein similarity network illustrating the excellent match of this sequence to fuconate dehydratase sequences is provided in Figure 6.

The sequence gi: 71915096 (GenBank:AAZ54998) is an example of an MFR misannotation from the enolase superfamily. Although it was annotated in NR as an *o*-succinylbenzoate synthase (OSBS) and scored against the HMM for that family, the general base required for catalysis of the enzymatic reaction, lysine 166, is substituted in this sequence with a histidine. This sequence also contains a number of additional substitutions in sequence motifs conserved in authentic members of the OSBS family [48]. Glasner et al. have discussed this protein in depth and enumerate the reasons (including genome context) why this sequence likely represents a new and unknown function in the enolase superfamily, rather than an OSBS.

The sequence gi 16082480 (GenBank:NP_393564) provides an example of the BTC type of misannotation. This sequence was annotated in NR as galactonate dehydratase. It scored against the galactonate dehydratase family HMM at a bit score of only 126.6, well below the TC for this family, 843.6, and was therefore classified as misannotated. Additionally, the sequence scored well against the gluconate dehydratase family HMM. The gluconate dehydratase family was not one of the 37 families used as a gold standard in this study because insufficient experimental information was available in the SFLD when our analysis was performed. Additional alignment and operon context information is now available to predict that gi 16082480 is indeed a gluconate dehydratase rather than a galactonate hydratase (see the SFLD).

The detailed results from this study are available in Dataset S1 or from the authors. Further work is underway to provide these results online as part of a “misannotation resource” at the SFLD web site (http://sfld.rbvi.ucsf.edu).

## Discussion

The misannotation levels determined in this work are substantially higher than those reported in previous studies. Several reasons may account for these high levels. First, this study is different in methodology from earlier studies that estimated levels of misannotation in specific genome projects. Two important earlier studies that predicted misannotation levels did so based on discrepancies in annotations made by different groups for specific genomes (for example, [4],[5]), allowing placement only of a lower limit on the likely levels of misannotation. In this study, precise levels of misannotation could be documented for specific sequences using a set of experimentally characterized families as a gold standard. Second, in the archival databases NR and TrEMBL, annotations are still largely made by inference from simple sequence similarity, arguably the least accurate approach for annotation transfer still in use [49]. Thus, misannotations in these resources might be expected to be relatively high. Third, most of the investigations focused on misannotation were published early in the genomic era [4],[5]. Our study is not unique in finding increased levels of misannotation relative to earlier studies [12]. Finally, the families and superfamilies evaluated here likely represent somewhat more challenging problems for annotation than do many groups of proteins for which ortholog prediction is straightforward. This is because each superfamily in our test set contains multiple homologous families that catalyze different chemical reactions – yet all of the families in each superfamily share a conserved chemical capability supported by conserved active site motifs [50]. This complicates annotation transfer based on simple approaches such as annotation transfer from the best match to a previously annotated sequence. Besides those used in our study, many additional enzyme superfamilies have now been identified that contain multiple families that catalyze different chemical reactions (see [51] for a compilation of some of these). Misannotation levels in many of these additional superfamilies (and new outlier enzymes identified in genomic and metagenomic projects [52]) may also be especially high, although our results cannot be broadly generalized to all annotations in the public databases we investigated.

Another key observation from this study is the high variability in the level of misannotation across both superfamily and family test sets (Figure 3 and Table S1). Several issues were examined that could account for this variability. Previously, others have shown that annotation transfer at low levels of similarity greatly increase the likelihood of incorrect function annotation [53]–[55]. Unfortunately, the records available at NR are insufficient to determine the specific routes by which sequences have been annotated so that it is not possible to explicitly determine whether most misannotation is the result of annotation transfer at low levels of similarity. We were able to examine whether misannotation was more prevalent in families with greater sequence diversity (i.e. families with low average pairwise percent identity). The average and range of pairwise percent identity for each of the 37 families in our gold standard set were calculated and the results showed no correlation between sequence similarity and the levels of misannotation (data not shown). Other characteristics, including superfamily size and family size (i.e. the number of sequences correctly annotated to each) also failed to show correlation with misannotation levels. We speculate that the variability in misannotation levels seen in this study is likely associated with many factors associated with the unsystematic way in which sequences have been annotated in these databases [56]. Many problems with annotation approaches, including annotating based only on similarity to the nearest neighbor, failing to adequately account for multidomain proteins and annotating based on inappropriate levels of sequence similarity have been discussed by others [2],[3],[57]. To accommodate this variation we suggest that thresholds for homology-based annotation transfer should be determined in a family-specific manner. Based on related observations, this suggestion has been previously made [54].

Examination of misannotation levels over time suggests that error propagation, likely occurring in a complex manner and associated with multiple methodological causes, is also a primary cause of the high and varied levels of misannotation that were observed. To examine qualitatively the possibility of error propagation, we modeled the emergence of misannotations over time using protein similarity networks. An all-by-all BLAST [58] was performed on all the sequences analyzed from the 37-family test set in the NR database and the results were visualized as a network [59],[60] (Video S1). The movie shows that as time progresses from 1993–2005, single proteins misannotated at early dates often became connected at later dates by new edges to sequence-similar proteins with the same incorrect annotations. A possible interpretation of this result is that these clusters of misannotated proteins emerged from error propagation from a single similar sequence that was misannotated early in the time period covered by this analysis. The expected continued use of simple annotation transfer for functional annotation for sequences submitted to the NR and TrEMBL databases from large scale sequencing projects suggests that this trend is likely to continue [39]–[41].

### Misannotation issues for large-scale databases

Two other primary issues for databases that are annotated largely by automated methods deserve discussion: the common use of source information without adequate reference and the inability to correct misannotated functions. These appear to contribute to the especially high levels of misannotation found in the archival databases NR and TrEMBL, and, we speculate, by transfer of information from these databases to KEGG.

A specific example from this study highlights the use of information from sites such as Pfam [31] or InterPro [32] to provide annotations without sufficient reference back to these resources. This practice is problematic because the original source information from such sites may not actually refer to a specific functional annotation but rather may only refer to a group of proteins sharing the same structural domain but representing multiple different functions. Frequently, however, the qualifying designation of ‘domain’ or ‘superfamily’ is not included in the final annotation, leading a user to conclude that such broad annotations represent specific functions. For example, in this study it was found that the description ‘mandelate racemase/muconate lactonizing enzyme’ was used to annotate some members of the enolase superfamily in the NR, TrEMBL and KEGG databases without identification of the source. This annotation is problematic because it is in fact the descriptor of the N- and C-terminal Pfam-A models of the same name (PF01188 and PF02746) that include many different enolase superfamily functions [44]. To a user unfamiliar with this superfamily, this annotation appears to describe a multifunctional enzyme that performs both racemization and lactonization reactions. Such a multifunctional enzyme in the enolase superfamily does not exist. We assume that this annotation was originally meant to indicate membership in a subgroup of related proteins in this superfamily that was defined by Pfam. Although this example could be considered as a type of misannotation likely to cause considerable confusion for users, it was not counted in the misannotation levels given in Figure 3 since it did not fall within the protocol used in this study.

A direct consequence of the use of source information without proper attribution is that it becomes essentially impossible to propagate corrections for misannotated sequences either back to the original source of the annotation or to secondary sources to which these annotations have been propagated. The glyoxalase I example given in Table 1 is a case in point, underscoring the difficulty of back propagating corrected annotation information through a database to sequences and annotations that are already there. As has been described elsewhere [61],[62], correcting misannotation in large archival databases such as NR is usually not possible because NR is not just a database but is also an archival library for which a primary mission is to keep an accurate record of sequence submissions, along with author supplied annotations. Thus, NR is not the owner of its annotations (or misannotations); rather, they are owned by the author(s) or genome sequencing project that submitted them.

### Addressing the issue of misannotation

Misannotation of molecular function in public databases continues to be a significant problem, particularly when new annotations are made by annotation transfer based on similarity, increasing the urgency for alternative strategies for obtaining high-confidence annotations. Discussion of whether and how to reannotate sequences for which incorrect annotations are already embedded in primary (and secondary) databases is an active topic in the literature [17], [18], [61]–[63]. We offer here suggestions that could be implemented to alleviate some of the worst consequences of the problem going forward.

First, we advocate, as many others have suggested, the use of evidence codes to provide attribution of the evidence used in support of a particular annotation. A growing number of databases including Swiss-Prot and the SFLD have added evidence codes for this purpose and evidence codes are integral to the GO effort as well [64],[65]. In this work, we created “misannotation evidence codes” to label the type of misannotations found. Evidence codes are useful because they convey important information simply and clearly. They are also readable by computers, thereby facilitating automated analyses by providing systematic definitions for evidence supporting an annotation. For example, to find all sequences that have been experimentally characterized in a database, e.g., in GO, one can simply filter the database by the evidence code “Inferred from Direct Assay” (IDA) and quickly retrieve the sequences of interest. Evidence codes require little effort to add to an annotation when it is originally generated and should generally be incorporated as a structured element of these records. (We note here that some annotations for test sequences examined in this study were modified by what might be considered qualifying evidence. These included terms that modified functional designations such as “hypothetical,” “predicted,” and “likely.” Although perhaps intended for use as a type of rudimentary evidence code, their meanings are not defined precisely, nor are they systematic in ways easily classified by computers. For these reasons, these terms were ignored in this study.)

In addition to simple operational ideas such as evidence codes to improve annotation quality and utility, our results showed that a major source of misannotation can be ascribed to “over annotation” of function, a concept that has also been described by others [57],[63]. The majority of misannotated sequences identified in this study (85%) resulted from over annotation, i.e., sequences were annotated with a specific family function even though they scored well only to a superfamily HMM but not to a family HMM. Thus, we suggest a more conservative approach to annotation, i.e., annotation only at the level of function for which there is strong evidence [45],[46]. If available evidence allows clear placement of a sequence only to a superfamily but not to a family within it, it should only be annotated as a member of that superfamily. This strategy is used by the SFLD, which annotates sequences at different levels of granularity based on supporting evidence for annotation to each, allowing us to claim high confidence annotations for this manually curated database.

The consequence of this approach is that many sequences would be annotated with only general functional characteristics common to all members of an enzyme superfamily, lowering significantly the number of sequences for which reaction specificity is annotated. Clearly, there is a tradeoff between the value of annotating most sequences with some level of function to facilitate interpretation of genomes and the confusion and misinterpretation that may result as misannotations continue to accumulate at a high levels. Undoubtedly, the individual scientist must choose where along the “annotation confidence” spectrum is most appealing for a particular study.

Our results also highlight the value of building and supporting manually curated databases that rely heavily on experimental evidence available from many types of biological experiments. These include Swiss-Prot as well as some organism-specific databases and specialty databases such as the Catalytic Site Atlas [28] and the SFLD. However, a main drawback to manual curation is the difficulty of keeping pace with new functional data resulting in far smaller and less representative databases than their automatically curated cousins [66]. Additionally, databases can be subject to bias because of the punctuated nature of scientific work, i.e., some protein functions and sequences attract much scientific attention, investigation and publication while others attract very little. As has been pointed out previously [53], Swiss-Prot exhibits such bias in its ‘overrepresentation’ of the sequences about which the scientific community knows most (contributing to the high quality of these annotations). Still, given the considerable difference in quality between manually and automatically curated databases, additional focus and resources should be devoted to manual curation. For example, the scientific community might consider enabling the submission of all experimentally characterized sequences to a centralized source such as Swiss-Prot at the time of publication, enhancing its size and currency. Such an effort could also contribute to broadening representation of the protein universe in these manually curated databases.

### Conclusion

This study examined the incidence of misannotation for over 7,000 sequences from the major archival databases and documents its prevalence, major types and some of its causes. Using a gold standard test set of well characterized enzyme families and superfamilies representing 5 fold classes, and spanning five of the six major classes of the E.C. system, our results demonstrate similar and surprisingly high levels of misannotation across all of the databases evaluated, except for Swiss-Prot, which showed very low levels of misannotation overall. We additionally found considerable variation in levels of misannotation across each of the 37 families examined. This result suggests that it will likely be difficult to predict even relative levels of misannotation for other superfamilies and families generally without the careful analysis of each. How our conclusions apply to other classes of proteins besides enzymes cannot be determined from this study. However, based on the breadth of the test set we investigated, we expect misannotation in public databases, at least for other functionally diverse enzyme superfamilies, to be a larger issue than has previously been estimated.

We found evidence for error propagation and an increase in annotation errors over time, indicating that the problem is getting worse even as multiple orthogonal information sources and tools are becoming available to complement simple annotation transfer protocols. Several major types of misannotation were identified, with a large majority (85%) associated with “over annotation,” i.e., annotation of sequences at a greater level of functional specificity than available evidence supports. We suggest that support for manually curated databases, including organismal databases and databases such as Swiss-Prot, could provide high confidence annotation for a subset of proteins. For large databases annotated largely by automated methods, the misannotation problem could be ameliorated to some extent by the use of evidence codes describing in a systematic and computer-readable format the evidence available to support annotation assignments.

## Methods

### Selection of functions to investigate for misannotation

The functions analyzed in this investigation were selected from the August 11, 2005 version of the Structure-Function Linkage database (SFLD) [46]. A set of functional families was defined for use as a gold standard, each of which met two criteria: catalytic residues needed for enzymatic function had been identified from experimental studies, and suitable manually curated hidden Markov models [67] and alignments were available. In all but two cases (galactonate dehydratase and 3-hydroxyisobutyryl-CoA hydrolase) at least one x-ray crystal structure was also available for each family. Superfamilies and families from the SFLD have been previously described as a Gold Standard for use as a benchmark for the development of computational tools for function prediction [44]. The set of enzymes used here are a subset of families from that work plus families from the terpene cyclase superfamily, which were added to the SFLD after the publication of the Gold Standard set. These families come from six superfamilies representing five different structural folds and 37 different functional families (Figure 1): enolase superfamily (10 families), crotonase superfamily (6 families), vicinal oxygen chelate superfamily (4 families), terpene cyclase superfamily (6 families), haloacid dehalogenase superfamily (4 families) and amidohydrolase superfamily (7 families).

### Protein sequence data

Four public databases were analyzed for misannotation: the NCBI GenBank Non-redundant (NR) protein database [1] the UniProtKB/TrEMBL and UniProtKB/Swiss-Prot protein databases [42] and the protein database of the Kyoto Encyclopedia of Genes and Genomes (KEGG) [43]. The protein sequences from these four databases were downloaded on February 17, 2006. Additionally, the protein sequences from the Gene Ontology (GO) database [64] were downloaded on the same day. These sequences were used as ‘knowns’ in the definition of family threshold (see below).

### Misannotation analysis protocol

#### Keyword search

The misannotation analysis followed the protocol given in Figure 2 and was identical for each database examined and family analyzed. A keyword search was used to gather sequences from the test databases. Keyword dictionaries were created for each family using information available from the SFLD and, when appropriate, the functional information and synonym lists from the Enzyme Commission (EC) [47]. Sequences with annotations matching one or more of the keywords were retrieved using regular expression string matching.

#### Misannotation prediction and classification

Sequences retrieved by the keyword search were scored in an automated fashion against all of the HMMs in the SFLD using the HMMER program hmmpfam A highly permissive and inclusive E-value cutoff of 100 was used for this step to gather highly divergent hits and to determine at what scores sequences from related families hit each family HMM. Using hmmalign (HMMER) each sequence was aligned to each HMM it scored against and discrepancies between the sequence and residues known to be necessary for catalysis were output.

#### Initial text parsing

The annotation of every sequence retrieved was examined manually. The sequences associated with annotations unrelated to the analysis function or that were not annotated to an enzymatic function (including sequences annotated only to a gene name) were removed. If an annotation contained both an enzymatic designation and a designation not associated with its catalytic functionality (e.g. localization or biological role) only the catalytic designation was analyzed. Annotations that used the terms ‘family’, ‘-like’, ‘similar to’, ‘related to’ and ‘homolog’ were not included in the final analysis set. Terms like ‘family or ‘homolog’ do not denote a specific reaction and can be inferred to mean either similarity in function or similarity in sequence based upon the user's context. As there was no specified context for these terms in the annotations, it was not possible to disambiguate the ‘functional similarity’ annotations from the ‘sequence similarity’ annotations, therefore, all such annotations were removed. The descriptors of ‘hypothetical’, ‘probable’, ‘putative’, ‘potential’, ‘predicted’ and ‘likely’ are also not well-defined terms [15] and serve only as qualifiers of unknown strength regarding the confidence of a functional prediction. These qualifiers were not considered in the analysis, i.e., annotations that included these types of descriptors were analyzed as though they were not present. If an annotation contained both a general description and a specific description (e.g. “(GLYOXALASE I HOMOLOG); lactoylglutathione lyase”) only the portion of the annotation that defined a specific function was analyzed. Additionally, fragments were removed from the analysis. A sequence was considered a fragment if it was too short either at the N or C terminus to contain all functionally important residues. A sequence that by alignment appeared to be missing interior portions of sequence was not considered a fragment. Sequences associated with crystal structures that had been mutated to remove required catalytic residues were not included in the test set. The sequences that remained after these steps constituted the analysis set.

#### Manual misannotation analysis steps

Using the output from the automated HMMER-based analysis, pruned as described, each sequence in the analysis set was analyzed in a four-step process and labeled with appropriate misannotation codes if a misannotation was found (Figure 2). The first step determined whether a test sequence mapped to the appropriate superfamily. If the sequence did not score against the appropriate superfamily HMM, it was labeled as misannotated and was classified with the ‘No Superfamily Association’ (NSA) misannotation code. The second step was to determine if the sequence under examination mapped to the appropriate family. If the sequence did not score against the family HMM to which it was annotated, the sequence was labeled as misannotated and classified as ‘Superfamily Associated Only’ (SFA). In the third step it was determined whether the sequence under examination contained the amino acid residues necessary for catalytic function. If the output of the automated alignment of the sequence against the family HMM indicated a discrepancy for the residues in question, additional manual analysis was undertaken (see below). Sequences missing one or more of functionally necessary residues were labeled as misannotated and classified as ‘Missing Functionally Important Residue(s)’ (MFR). Conservative amino acid substitutions and/or mutations that might still be functional (e.g. likely able to bind to a metal etc.) were accepted, however. The fourth step was used to determine if a test sequence scored sufficiently well against to the family HMM to be considered a true family member. This was determined by a threshold named the Trusted Cutoff (see the section describing threshold definitions below). If a sequence did not score above the Trusted Cutoff, it was labeled misannotated and classified as “Below Trusted Cutoff’ (BTC). Any sequence that passed all four of these steps was considered to be annotated correctly. In total, over all four databases, 7255 sequences were examined in the misannotation analysis (Figure 1); Annotation designations are provided for these sequences in Dataset S1.

#### Assessment of functional residue designation

Every sequence that was found by the automated process to be missing one or more functionally important residues was checked manually. First, the alignment of the sequence to the family HMM alignment was visually inspected to ensure that there was no obvious misalignment or conservative substitution (conservative amino acid substitutions were accepted). Using the alignment program Muscle [68], sequences with non-conservative substitutions of functionally important residues were aligned to the sequences of authenticated family members. The alignments were manually analyzed, checked against available literature and case-by-case decisions were made whether to accept these non-conservative substitutions. Sequences for which the mutations were accepted were passed on to the next (and final) analysis step (threshold step, Figure 2).

### Thresholds to determine family (functional) membership

In order to differentiate family members from non-family members, HMM bit-score thresholds were determined for each gold standard family. Sequences in the SFLD assigned to families and sequences from GO that were marked with the evidence code “Inferred from Direct Assay” (IDA) were scored against all of the SFLD HMMs using the HMMER program hmmpfam using an E-value cutoff of 100 (14,902 sequences scored). The scores were compiled and the sequences labeled according to whether they were true positives or true negatives for the family against which they scored. The Trusted Cutoff (TC) was defined as the HMM score of the lowest-scoring true family member against the family HMM (Figure S1). The TC was the threshold at which the primary misannotation analysis was performed.

### Change in misannotation over time

For each sequence analyzed in the GenBank NR database, the original submission date of that sequence was retrieved from NR. The sequences were binned by submission year and predicted annotation status (Figure 3). The fractions of predicted misannotated sequences versus the total number of sequences deposited were calculated as follows: misannotated sequences deposited in year X/total sequences deposited in year X.

### Protein similarity networks

To generate the network shown in Figure 6, an all-by-all BLAST analysis of sequences of a subgroup of families from the enolase superfamily was performed. A protein similarity network [60] was created from the BLAST results using the software Cytoscape [59]. The nodes were arranged using the yFiles organic layout provided with Cytoscape version 2.4. Connections between nodes were shown as edges if the E-value of the best BLAST hit between two sequences is at least as good as 1×10^−50^ (As these BLAST analyses were performed using a custom sequence database the resulting E-values are not necessarily directly comparable to the E-values determined by BLASTing against databases with large background models such as GenBank NR [60]). Tools used for visualization of protein networks were created in part by the UCSF Resource for Biocomputing, Visualization, and Informatics, supported by NIH P41 RR-01081, and are available from the Resource (http://www.rbvi.ucsf.edu).

### Data plots

All data plots were produced using the software R v2.6.0.

## Supporting Information

Figure S1 Three analysis thresholds used in the misannotation analysis. This example for the galactonate dehydratase family (enolase superfamily) illustrates how the three scoring thresholds were defined for each of the 37 families evaluated in this study. The Trusted Cutoff (TC) (used for the primary misannotation analysis) was defined as the lowest score at which a true family member scores against the family HMM. The Noise Cutoff (NC) threshold was defined as the highest score at which a non-family member scores against the family HMM. The Lenient Cutoff (LC) threshold uses the set of true family sequences to which some false positive sequences have been added so that they represent 5% of the total sequences. Using this artificial set of family sequences, the LC threshold for each family was defined as the lowest score at which one of these non-family sequences scored.
(1.00 MB TIF)

Figure S2 Average percent misannotation in the NR database across families in each superfamily using different thresholds. The black bar in each plot depicts the average percent misannotation predicted in the analysis over each superfamily at the three scoring thresholds described in additional figure 1.
(0.01 MB TIF)

Table S1 Percent misannotation for each family in the NR, TrEMBL, KEGG and Swiss-Prot databases.
(0.13 MB DOC)

Text S1 Misannotation analysis controls and tests
(0.06 MB DOC)

Dataset S1 Sequences analyzed in misannotation analysis and their designations
(1.21 MB XLS)

Video S1 Movie of the annotations from the NR database displayed by year (1993–2005). The movie tracks correctly annotated and misannotated sequences in the test set over the years 1993–2005. The similarity network is arranged by superfamily and colored as in figure 1, i.e. all nodes of the same color were annotated to the same function. The network was generated from an all-by-all BLAST analysis of the test sequences with results that had BLAST E-value scores of 1×10−30 or lower retained. Nodes represent sequences deposited into the NR database during the years 1993–2005. Any two nodes are connected by an edge if at least one node found the other with a BLAST E-value less than or equal to 1×10−30. The network is visualized using Cytoscape v2.6.0-beta. The distance between any two connected nodes is roughly inversely proportional to the strength of the E-value between them (force-directed layout). The shapes of the nodes indicate annotation status: circles depict correctly annotated sequences and triangles depict incorrectly annotated sequences. Black arrows indicate examples in the haloacid dehalogenase family (HAD) and glyoxalase I family (VOC) that display potential evidence of error propagation. As these BLAST analyses were performed using a custom sequence database the resulting E-values are not necessarily comparable to the E-vaules determined by BLASTing against databases with large background models such as GenBank NR [60].
(1.29 MB MOV)
