# Supplementary figures and images for: Annotation Error in Public Databases: Misannotation of Molecular Function in Enzyme Superfamilies
Source: PLoS Comput Biol. 2009 Dec 11;5(12):e1000605. doi: 10.1371/journal.pcbi.1000605 (PMC2781113; doi:10.1371/journal.pcbi.1000605)

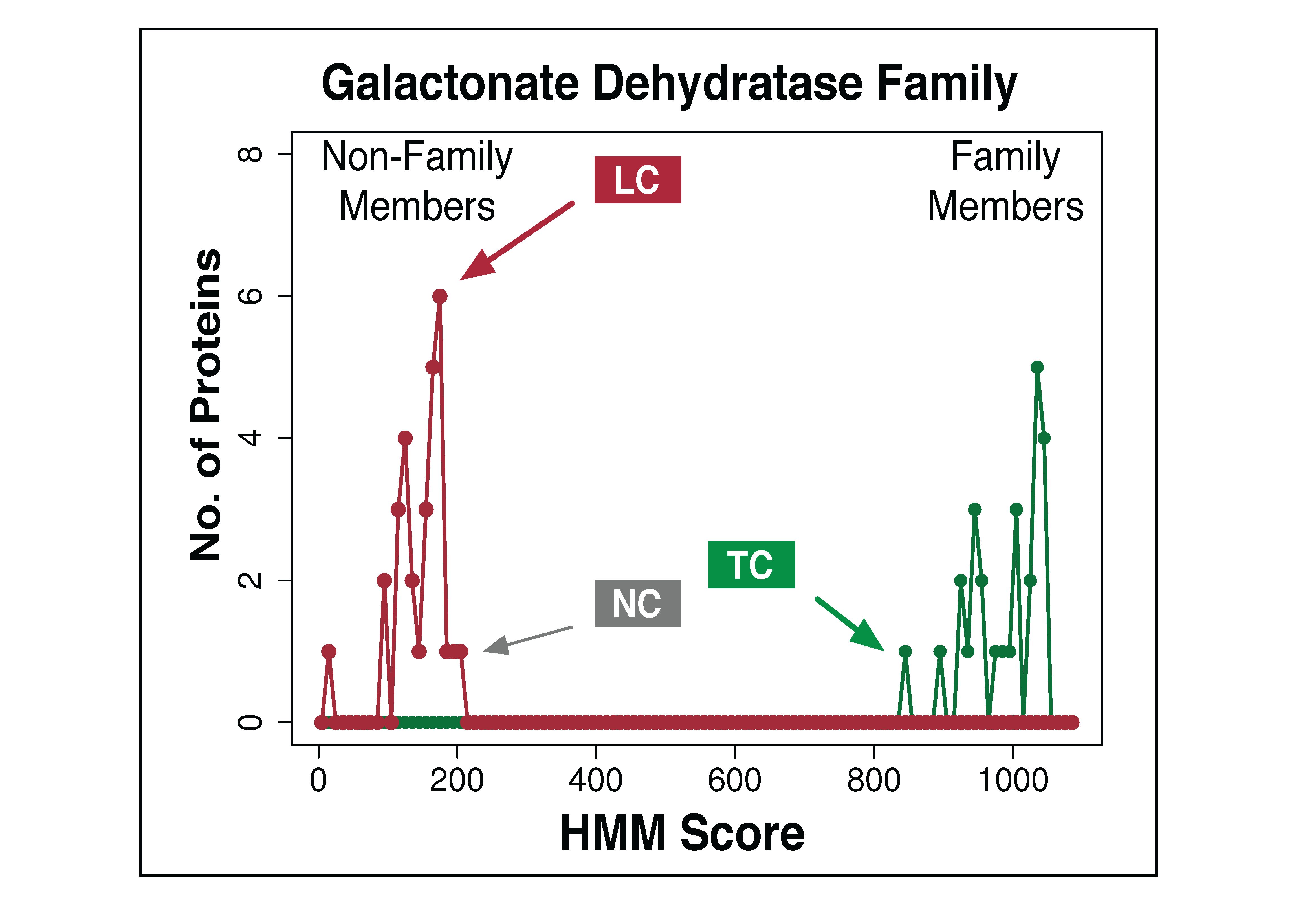

Supplement: Figure S1 — Three analysis thresholds used in the misannotation analysis. This example for the galactonate dehydratase family (enolase superfamily) illustrates how the three scoring thresholds were defined for each of the 37 families evaluated in this study. The Trusted Cutoff (TC) (used for the primary misannotation analysis) was defined as the lowest score at which a true family member scores against the family HMM. The Noise Cutoff (NC) threshold was defined as the highest score at which a non-family member scores against the family HMM. The Lenient Cutoff (LC) threshold uses the set of true family sequences to which some false positive sequences have been added so that they represent 5% of the total sequences. Using this artificial set of family sequences, the LC threshold for each family was defined as the lowest score at which one of these non-family sequences scored. (1.00 MB TIF) [file pcbi.1000605.s001.tif]
