# Supplementary material for: Annotation Error in Public Databases: Misannotation of Molecular Function in Enzyme Superfamilies
Source: PLoS Comput Biol. 2009 Dec 11;5(12):e1000605. doi: 10.1371/journal.pcbi.1000605 (PMC2781113; doi:10.1371/journal.pcbi.1000605)
Supplement: Table S1 — Percent misannotation for each family in the NR, TrEMBL, KEGG and Swiss-Prot databases. (0.13 MB DOC) [file pcbi.1000605.s003.doc]

| Superfamily | Family | NR % Mis. | | | TrEMBL % Mis. | KEGG % Mis. | Swiss-Prot % Mis |
| --- | --- | --- | --- | --- | --- | --- | --- |
|  |  | Thresh.^a^ | | | Thresh. | Thresh. | Thresh. |
|  |  | TC^b^ | NC^c^ | LC^d^ | TC | TC | TC |
| Enolase |  |  |  |  |  |  |  |
|  | Enolase | 4 | 3 | 3 | 4 | 2 | 0.4 |
|  | Galactonate dehydratase | 32 | 26 | 26 | 36 | 38 | 0 |
|  | Mandelate racemase | 90 | 90 | 68 | 100 | 100 | 0 |
|  | Glucarate dehydratase | 30 | 26 | 10 | 32 | 40 | 20 |
|  | Methyl aspartate ammonia-lyase | 52 | 27 | 0 | 65 | 63 | 0 |
|  | Ortho-succinyl benzoate synthase | 5 | 5 | 5 | 7 | 7 | 0 |
|  | Dipeptide epimerase | 50 | 50 | 50 | 100 | 0 | 0 |
|  | Chloromuconate cycloisomerase | 69 | 69 | 69 | 67 | 100 | 17 |
|  | Muconate cycloisomerase | 55 | 52 | 52 | 54 | 74 | 0 |
|  | L-fuconate dehydratase | 0 | 0 | 0 | 0 | 0 | 0 |
|  | Superfamily Avg. % Misannot. | 24 | 22 | 18 | 22 | 22 | 1 |
| Crotonase |  |  |  |  |  |  |  |
|  | Dodecenoyl-CoA delta-isomerase (mitochondrial) | 0 | 0 | 0 | 100 | 59 | 0 |
|  | Delta(3,5)-delta(2,4)-dienoyl-CoA isomerase | 10 | 10 | 10 | 0 | 100 | 0 |
|  | Methylmalonyl-CoA decarboxylase | 0 | 0 | 0 | 0 | 0 | 0 |
|  | 3-Hydroxyisobutyryl-CoA hydrolase | 19 | 13 | 6 | 19 | 22 | 0 |
|  | 4-Chlorobenzoate dehalogenase | 0 | 0 | 0 | 0 | 0 | 0 |
|  | 1,4-Dihydroxy-2-napthoyl-CoA synthase | 0 | 0 | 0 | 0 | 0 | 0 |
|  | Superfamily Avg. % Misannot. | 12 | 8 | 4 | 32 | 46 | 0 |
| VOC |  |  |  |  |  |  |  |
|  | Methylmalonyl-CoA epimerase | 77 | 55 | 0 | 88 | 75 | 0 |
|  | 4-Hydroxyphenylpyruvate dioxygenase | 18 | 9 | 8 | 17 | 14 | 4 |
|  | FosA | 27 | 27 | 27 | 100 | 100 | 0 |
|  | Glyoxalase I | 71 | 60 | 42 | 72 | 75 | 0 |
|  | Superfamily Avg. % Misannot. | 52 | 42 | 29 | 57 | 61 | 2 |
| Terpene Cyclase |  |  |  |  |  |  |  |
|  | 5-Epi-aristolochene synthase | 9 | 9 | 9 | 0 | 0 | 0 |
|  | Bornyl diphosphate synthase | 0 | 0 | 0 | 0 | 0 | 0 |
|  | Pentalenene synthase | 0 | 0 | 0 | 50 | 50 | 0 |
|  | Squalene-hopene synthase | 10 | 5 | 5 | 9 | 0 | 0 |
|  | Trichodiene synthase | 1 | 1 | 0 | 0 | 0 | 6 |
|  | Aristolochene synthase | 0 | 0 | 0 | 0 | 0 | 0 |
|  | Superfamily Avg. % Misannot. | 5 | 2 | 2 | 8 | 3 | 4 |
| HAD |  |  |  |  |  |  |  |
|  | Deoxy-D-mannose-octulosonate 8-phosphate phosphatase | 57 | 7 | 3 | 84 | 66 | 38 |
|  | Phosphonoacetaldehyde hydrolase | 32 | 11 | 4 | 30 | 23 | 0 |
|  | 2-Haloacid dehalogenase | 72 | 60 | 44 | 71 | 81 | 0 |
|  | Beta-phosphoglucomutase | 64 | 50 | 38 | 63 | 64 | 0 |
|  | Superfamily Avg. % Misannot. | 63 | 39 | 28 | 65 | 66 | 15 |
| AH |  |  |  |  |  |  |  |
|  | Cytosine deaminase | 32 | 32 | 32 | 31 | 43 | 0 |
|  | Adenosine deaminase | 80 | 77 | 31 | 88 | 82 | 70 |
|  | N-acyl-d-amino-acid deacylase | 66 | 48 | 23 | 72 | 54 | 0 |
|  | L-Hydantoinase | 25 | 25 | 25 | 50 | 0 | 0 |
|  | D-Hydantoinase | 20 | 9 | 9 | 27 | 40 | 0 |
|  | Urease | 2 | 2 | 2 | 1 | 4 | 0 |
|  | Isoaspartyl dipeptidase | 62 | 62 | 8 | 64 | 32 | 0 |
|  | Superfamily Avg. % Misannot. | 40 | 37 | 18 | 44 | 45 | 37 |

^a^ Misannotation HMM analysis thresholds

^b^ Trusted Cutoff

^c^ Noise Cutoff

^d^ Lenient Cutoff
