# Supplementary material for: Annotation Error in Public Databases: Misannotation of Molecular Function in Enzyme Superfamilies
Source: PLoS Comput Biol. 2009 Dec 11;5(12):e1000605. doi: 10.1371/journal.pcbi.1000605 (PMC2781113; doi:10.1371/journal.pcbi.1000605)
Supplement: Text S1 — Misannotation analysis controls and tests (0.06 MB DOC) [file pcbi.1000605.s004.doc]

**Additional Text S1: Misannotation analysis controls and tests.**

***Examination of Sequences in NR and Swiss-Prot Predicted to be Misannotated*.** To verify the accuracy of the misannotation analysis results using an alternative to our protocol, a search was made for literature that might contradict our predictions. The NCBI and Swiss-Prot accession pages for each sequence labeled misannotated were manually examined and the accompanying references were inspected for experimental characterization. In total the references of 1155 sequences were manually examined. Of the 1112 and 43 sequences predicted to be misannotated in NR and Swiss-Prot respectively, only 4 sequences (3 in NR, 1 in Swiss-Prot) could be associated with a publication that contained experimental results that conflicted with our predictions. One additional sequence in NR was found to be incorrectly classified as misannotated based upon an extensive study of genome context and evolutionary relationships [1]. Another sequence was found to be incorrectly classified based upon a collaborator’s manuscript in progress (F. Raushel, personal communication). For each of these 6 sequences (and other sequences ≥ 99% identity), our predictions of misannotation were corrected and so did not contribute to the final level of misannotation predicted.

***Assessment of Newly Characterized Sequences*.** Another test of our method was performed by analyzing sequences of proteins that had been experimentally characterized since the data used for this study was downloaded from the sequence databases. Twenty-seven sequences belonging to 4 superfamilies: enolase (7 sequences), crotonase (2 sequences), terpene cyclase (2 sequences), and amidohydrolase (16 sequences) were examined using the misannotation analysis protocol (figure 2). Of these, 26/27 were labeled by the analysis protocol as correctly annotated, consistent with the experimental results. One sequence, a muconate cycloisomerase in the enolase superfamily, scored against the family HMM slightly below the Trusted Cutoff for that family and was therefore incorrectly classified as misannotated. This example shows that threshold choice did have some affect on our misannotation results (see below and additional table 1).

***Stability of annotations****.* We examined whether corrections had been made for sequences that we had predicted to be misannotated. Of the 1112 sequences in the NR database found to be misannotated by our analysis, the annotations of 111 of were examined (~10%, chosen randomly). These 111 sequences were downloaded from the February 2008 NR database and the annotations were manually compared to the annotations from our download in 2006. Of these, 107 of the annotations were unchanged, three had new corrected functional annotations and for one sequence there was no longer a functional annotation.

***Modelling analysis dependence on thresholds*.** To determine the extent to which the choice of family thresholds (TC) might influence the misannotation results, two other thresholds, the Noise Cutoff (NC) and the Lenient Cutoff (LC) were used. The NC was defined as the HMM score of the highest-scoring non-family member against a given family HMM (additional figure 1). The LC is a permissive threshold created by adding erroneous data. This threshold was defined score at which 5% of the total sequences included were non-family members that scored best against the family HMM. The designation of the TC and NC thresholds is very similar to the process used by Pfam[2, 3].

There were several cases in which no non-family members scored against certain family HMMs. For these cases the LC was defined as a score of 40, which in our models, roughly corresponded to an E-value of 1x10^-10^. An E-value of 1x10^-10^ is often used as a lower bound threshold for retrieving true homologous sequences [4]. The NC was then defined as the midpoint between the TC and the LC.

In one instance, the glucarate dehydratase family (enolase superfamily), scores for non-family members and family members overlapped. In this case, the TC was defined as the lowest-scoring family member that scored above all non-family members. The NC was defined as the score at which the first non-family member scored. The LC was then defined as the lowest-scoring family member. This score was lower than the score that would have been determined for the LC by simply adding erroneous sequences (the normal method to determine the LC).
